# Factors Generating the Willingness of Romanian Consumers to Buy Raw Milk from Vending Machines

**DOI:** 10.3390/foods12112193

**Published:** 2023-05-30

**Authors:** Marius Mircea Sabău, Pompei Mititean, Cristina Bianca Pocol, Dan-Cristian Dabija

**Affiliations:** 1 Department of Economic Sciences, University of Agricultural Sciences and Veterinary Medicine of Cluj-Napoca, 400372 Cluj-Napoca, Romania; marius.sabau@usamvcluj.ro; 2 Department of Accounting and Audit, Faculty of Accounting and Management Information Systems, Bucharest University of Economic Studies, 010374 Bucharest, Romania; mititeanpompei19@stud.ase.ro; 3 Department of Animal Production and Food Safety, University of Agricultural Sciences and Veterinary Medicine of Cluj-Napoca, 400372 Cluj-Napoca, Romania; 4 Department of Marketing, Faculty of Economics and Business Administration, Babeș-Bolyai University, 400591 Cluj-Napoca, Romania

**Keywords:** vending machines, willingness to buy, stimulus–organism–response (SOR) approach, milk dispensers, raw milk, short food supply chain, Romania

## Abstract

The use of automatic raw milk dispensers for products obtained from Romanian farms can represent an effective method of encouraging the development of short supply chains and promoting sustainable production and consumption systems. There are very few studies in the literature, especially in emerging economies, that analyze consumer perception regarding the use of raw milk dispensers; most of the research is focused on technical aspects regarding how such machines function and food safety, and less on consumers’ perceptions towards them or consumer satisfaction, loyalty, or intention to use them. Therefore, the objective of this research was to investigate the willingness of Romanian consumers to buy raw milk from vending machines. In this regard, the authors drew a conceptual model to assess the factors that trigger willingness to buy raw milk from vending machines and then implemented a quantitative-based survey among Romanian consumers who buy raw milk from vending machines. The data were analyzed by modeling structural equations with SmartPLS. The results reveal that the generation of consumer willingness to buy raw milk from vending machines depends on how consumers perceive raw milk but also on the product safety, reusability of the milk bottle, and the provenance of the raw milk, as well as the nutritional qualities of the unprocessed raw milk. The paper extends previous studies based on the stimulus–organism–response (SOR) and deepens the understanding of consumers’ perception towards raw milk dispensers. Furthermore, the results also highlight possible managerial strategies that aim to improve the understanding of consumers.

## 1. Introduction

Vending machines originated in 1888, over time becoming increasingly sophisticated and innovative, which allowed for the diversification of the types of products sold, packaging used, and payment methods [1]. This sector has registered significant growth in recent years, with Italy being the European leader in the production of such equipment, as well as the country that has the largest number of vending machines [2]. However, in the agricultural sector, they are not frequently used, except in a few cases, such as milk vending machines (the most popular) [3,4], bread machines [5], egg machines [6], fruit machines [7], and vegetable machines [8]. Unfortunately, many vending machines on the market dispense products in the ‘junk food’ category, which has given them a bad reputation [2]. However, their reputation increased during the sanitary crisis caused by the Coronavirus [7] but also because of interventions to stimulate a healthy lifestyle through the introduction of milk, fruits, and vegetables in schools [8,9,10,11]. Vending machines might also be classified as ‘healthy’ or ‘unhealthy’ [9]. 

Milk vending machines use the production of farms in the vicinity of their location, which contributes to the elimination of intermediaries between producers and consumers, that is, the development of short distribution circuits and business models specific to the circular economy [12,13]. At the same time, these milk vending machines play a role in reducing the use of resources and energy for packaging and transport, which is considered a suitable method for the efficient distribution of production, exerting a relatively low impact on the environment [12]. However, from the perspective of carbon emissions, the environmental impact assessment must also consider the amount of milk transported and the distance travelled between the milk’s production and the point of sale. The use of milk vending machines can be advantageous for farmers as a viable alternative to classic distribution systems by increasing profit margins [14]. From the perspective of consumers, buying raw milk from vending machines can be more advantageous in price and more accessible given that these vending machines are operational non-stop [14]. By using short distribution chains, including milk dispensers, a strong connection is created between the farmer and the consumer that is based on trust, quality, transparency, and authenticity [15].

Given that product and food packaging represents the main source of paper and plastic consumption, amounting, only in Europe, to approximately 40% of the total amount of plastic packaging made to 50% for that of paper [16], contemporary society must identify suitable solutions to limit the consumption of these materials or even to reduce them by identifying ways to sell food and/or other goods that allow for packaging reuse.

Unlike online shopping, which represents a very well-developed and investigated distribution channel [17,18,19], food purchase from vending machines is a relatively poorly addressed topic in the literature, especially in Romania, with existing studies using secondary data sources at regional level [20] or based on specific case studies [21] or on aspects related more to food safety [22,23,24,25]. Vending machines provide easy access to a wide variety of fresh or processed foods, beverages, and/or menus, and they can be in shopping centers, block staircases, hospitals, public institutions, educational institutions, and/or other places where there is socioeconomic activity [26,27,28]. The main feature of vending machines is the convenience they offer, namely, the reduction of contact with the seller [7]. Of course, resorting to them brings increased safety to the act of sales, with food hygiene representing an extremely important element, especially for quickly or easily perishable foods [4]. Although studies on the features and necessity of using different vending machines are quite numerous in the scientific literature [29,30], there are few studies based on the analysis of consumer perceptions regarding the different automatic food dispensers, even more so regarding raw milk ones in Romania.

To cover this research gap, the authors resorted to the implementation of empirical research of a quantitative nature, carried out using a questionnaire-based survey. The research was implemented in an emerging market, Romania, where there is a growing interest in consumer segments for natural, raw, and minimally processed foods sold through short supply chains [29,30,31], even if the literature [32,33] shows that supermarket chains are increasingly offering fresh food, including milk.

Thus, this research aimed to determine the intention to buy raw milk from vending machines because of the synergistic action of different relevant stimuli for consumers: nutritional features of raw milk, convenience of raw milk for food, safety of raw milk for consumption, origin of raw milk, possibility of reuse of the packaging, and image of raw milk. Studying raw milk from a consumer perspective is of great importance and relevance in contemporary society, as vending machines allow for direct contact between producers and consumers, thus enabling the elimination of intermediaries from the milk supply chain, lower prices for raw milk, and better milk quality. Of course, milk sold through vending machines must come from local farmers, because the lack of processing does not allow its transport over greater distances. 

The paper is structured as follows: Section 1 contains the theoretical framework, where the authors present the theoretical approach of the paper, followed by the development of the hypothesis and the research model. Section 2 continues with the research methodology and discussions, while the paper ends with conclusions in Section 5, which contains the theoretical and managerial contributions of the paper, along with the limitations and future research perspectives.

## 2. Review of the Literature

### 2.1. Theoretical Framework: The Stimulus–Organism–Response Model

In the literature, consumer behavior patterns are explained using the (S–O–R) theory [34,35], where the external stimuli (S) that affect the organism (O), especially on a cognitive level [36] and emotional level [37], thus shaping behavior (R) [38,39]. Based on this theory, characteristics such as nutritious milk, reusable milk bottle, and raw milk are considered external stimuli (S) that have emotional implications (O) from the perspective of raw milk knowledge provenance (RMKP) and raw milk image (RMI), thus generating a consumer preference (R) in terms of willingness to buy raw milk (WBRM).

This research makes an essential contribution to the advancement of literature and the stimulus–organism–response (SOR) behavioral model, because the different stimuli (the features of raw milk, nutritional value of raw milk, convenience of raw milk, safety of raw milk for consumption, origin of raw milk, and possibility to reuse the packaging) determine the anchoring of the raw milk image in the minds of consumers (organism) and, thus, generates the intention to buy it (response). 

### 2.2. Hypothesis and Research Model Development

Today, packaging plays an important role in the manufacturing process, maintaining the quality of the products for a long period, with its main role being to prevent food deterioration while being environmentally friendly [40,41]. The use of adequate packaging materials and procedures to prevent food loss and to offer safe and healthy food products has been a key point of food packaging research [42,43]. The role of packaging helps consumers eat food how and when they want [44]. The best-valued attributes that food packaging must fulfil are ease of opening, resealability, packaging size, and packaging material transparency [45,46]. When choosing food, packaging plays a key role, as it helps prevent cross-contamination of sealed food, but it also leads to convenient manageability [47]. When using a reusable shopping bag, hygiene risks increase, which makes it necessary to double check the packaging before its repeated use [48].

The extrinsic properties of products play a very important role for consumers in their purchase decision [49]. Food provenance, traceability, and consumer confidence in nutritional characteristics and values are relevant elements that contribute to preference for certain foods [50]. The purchase of milk in vending machines is based on the consumer’s belief that this food is superior from the point of view of its quality than that sold through classic distribution chains, being natural and probably healthier [51]. Often, milk sold through such machines is minimally processed, having organoleptic properties favorable for immediate, quick consumption but also a shorter shelf life [52].

The origin of the products, as well as their ‘local’ character, are important elements for consumers who want to engage in sustainable consumption [53]. Therefore, the consumer’s confidence in local products is higher compared to that given to food processed in an industrial system [54]. Consumer preferences in choosing and knowing the origin of local products include nutritional information, quality characteristics, safety, and reliability of use but also the degree to which they are produced according to organic standards [55]. For consumers, the processes by which food was obtained, production systems used, conventional or organic [56], nutritional profile, and indications regarding its provenance are very important. The origin of food, as well as its attractiveness, contribute to its positive appreciation by consumers [57]. Based on these arguments, we postulate that:

**Hypothesis** **H1.** *Nutritious features of raw milk correlate with the reusable raw milk bottle.*

**Hypothesis** **H2.** *Nutritious features of raw milk correlate with the provenance of raw milk knowledge.*

Food should be packaged to facilitate its transport, avoiding any interaction with the environment (i.e., alteration [58]), but also to capture consumers’ attention and their determination to choose the product [59]. Packaging is often used once and then discarded, which has a negative impact on the environment and contributes to increased pollution [60]. Therefore, one solution to reduce pollution is to reuse packaging [16]. To reduce packaging pollution, it is important that consumers are also educated on how to reuse or collect packaging for recycling. The literature [61] highlights that plastic packaging is often underestimated by consumers regarding its reusability, while glass and biodegradable components are much more commonly preferred. Consumer preference for raw milk is highly dependent on their desire to feel a stronger connection with nature and the origin of food. At the same time, when consuming raw milk, consumers disapprove of its intensive processing, to a certain extent, but also the fact that some staple foods are transported over very long distances, thus increasing carbon emissions [62].

Packaging based on paper and cardboard is advantageous from an ecological point of view, while plastic and metal are more polluting [63]. The need to educate consumers regarding the use, reuse, and/or recycling of packaging is very important, as they fail to clearly distinguish biodegradable from reusable packaging [64]. The use of recyclable packaging allows consumers to make their consumption more efficient and greener [65]. Consumers are prepared to reuse packaging primarily according to their type and less according to their intrinsic features, namely, the nature of the food they protect [66]. Three out of five people believe that packaging reuse is more important than its recycling, and 85% of consumers would prefer to buy products in packaging that could be reused [67]. These aspects help both to increase the visibility of products on the market and contribute to more detailed knowledge of the origin of the food assortment chosen by the consumer. Although milk packaging can be reused for an automatic vending system, milk sold in stores is packaged in single-use containers, which contributes to increasing the need for recycling but can also generate pollution. Therefore, consumers who use milk vending machines show an increased predisposition to environmental protection and reduce the amount of packaging that requires recycling [12]. Therefore, we hypothesize that:

**Hypothesis** **H3.** *Reusable raw milk bottles correlate with raw milk knowledge provenance.*

**Hypothesis** **H4.** *Reused raw milk bottles correlate with the image of raw milk.*

The rapid development of supply chains and the role they play in the economy have raised concerns about food safety and quality. Therefore, organizations around the world have introduced quality (i.e., ISO 9001) and safety standards to protect consumers [68]. The literature has identified that both the concept of quality and safety are closely related to perception [69,70]. Consumer preference for food packaging also depends on the existence of food quality and safety certificates, that is, their credibility in the food system [71]. The decision to purchase a food depends, to a large extent, on its quality but also on its origin and the rigor of the manufacturing process. The elderly are especially sensitive to the provenance of the food they consume [72].

The literature [73,74] reveals that consumers visit local restaurants because the food is healthier, tastier, and of higher quality. At the same time, buying local food supports the community and local economy. Food safety and quality are perceived differently by consumers; some are more orientated towards consuming and purchasing local or indigenous products at the expense of those available in commercial chains or sold through stores [75]. Among the characteristics related to food quality, consumers value freshness, safety, nutritional characteristics, and price [76,77]. The increased preference for milk marketed through vending machines will be influenced by factors such as [4] price, availability of different milk types, hygiene of premises in proximity to the machine, traceability of the product, and the lack of food safety risks. Therefore, we posit that:

**Hypothesis** **H5.** *Raw milk features correlate with the provenance of raw milk knowledge.*

**Hypothesis** **H6.** *Raw milk features correlate with the image of raw milk.*

The local purchasing behavior displayed by consumers differs depending on sociodemographic, contextual, situational factors, knowledge, previous experiences, motivations, and/or the attitudes of the individual towards purchasing local products [78,79]. Even if local foods are perceived as more expensive, certain segments of consumers are still more inclined to buy them [78]. Often, foods whose origin is known are preferred over industrial foods, with the knowledge of the local producer influencing their sales [80]. Men are willing to pay more for local products [81], which are preferred, to a greater extent, by young people who identify more strongly with the region in which they live, believing that this contributes to its development [82]. Consumers are willing to pay a higher price for food whose origin they know [83], with the price premium sometimes being 15% higher than for other food [84].

Intention is also determined by the perception that some foods are of better quality [85]. Depending on the level of education of the consumers [86], their desire to buy fresh food may be even greater [87]. The literature highlights the fact that the image that consumers anchor on a food essentially contributes to their intention to purchase it [88,89]. Attitude and perceived behavioral control were significant predictors of intention to purchase ethically sourced food [90]. Basically, the more unique, attractive, and relevant the features an individual has anchored in their mind concerning a certain product [91], the stronger will be their intention to search for or purchase that product or recommend it to others. The higher the consumer’s expectations are concerning the features of a product that are natural or made from natural ingredients, the greater the willingness to buy it [92]. Thus, based on these arguments, we considered the following:

**Hypothesis** **H7.** *Raw milk knowledge provenance correlates with consumers’ willingness to buy raw milk.*

**Hypothesis** **H8.** *Raw milk image correlates with consumers’ willingness to buy raw milk.*

**Figure 1:**
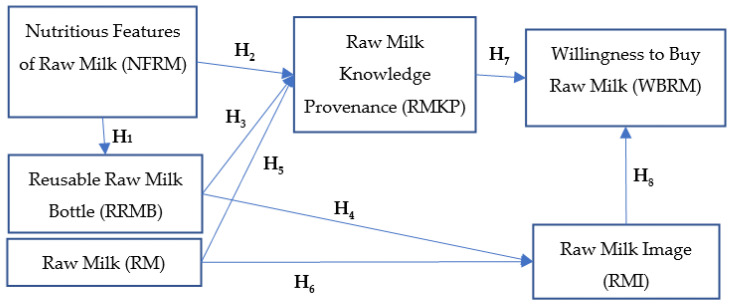
Conceptual model: generation of the willingness to buy raw milk from vending machines.

Based on the arguments presented, we propose the following conceptual model (Figure 1), which highlights the impact of the characteristics of milk on generating knowledge about this food and the determination of the intention to purchase raw milk sold through vending machines.

## 3. Research Methodology

### 3.1. Research Design

The investigation was carried out in an emerging market (Romania) where vending machine studies are very rare but where consumers still prefer minimally processed and natural foods [93]. The research was based on convenience sampling, because in the considered emerging market, there are no exact statistics on the number of consumers of raw milk sold through vending machines. A questionnaire was developed based on different scales extracted from the literature (see Table 1), using a five-point Likert scale (total disagreement/total agreement). The questionnaire was operationalized as follows: nutritious features of raw milk (NFRM), reusable raw milk bottle (RRMB), raw milk (RM), raw milk knowledge provenance (RMKP), raw milk image (RMI), and willingness to buy raw milk (WBRM). 

The authors performed an initial pre-test of the questionnaire by presenting it to different experts in the field, as recommended in the literature [94]. Minor adjustments were made to some of the statements so that the questionnaire could be distributed. Before filling in the questionnaire, the respondents were informed that when assessing the statements, they should think of the standardized milk vending machine they know best that sells raw milk from local farmers. All raw milk vending machines in the considered emerging market are standardized, having their own self-cleaning features and sensors that monitor different parameters of the contained milk [23]. The milk sold through such vending machines is raw, fulfilling the criteria of EU legislation comprising hygiene rules for food of animal origin [95]. Such vending machines always inform customers that milk must be boiled before consumption to minimize the transmission of viruses and diseases from cows to humans. 

The authors distributed the questionnaire to various social media groups of consumers who prefer minimally processed and/or unprocessed foods, inviting their members to respond to the research and to distribute the questionnaire to other interested people. Thus, the objective was to obtain a snowball effect [96]. Data were collected between 2021 and 2022, with relative difficulty due to the COVID-19 pandemic in the rapid implementation of research.

**Table 1:** Scale reliability.

Construct	Item	Measure
Nutritious Features of Raw Milk (NFRM), adapted from [55,97]	NFRM1	Raw milk comes from a local farm.
	NFRM2	Raw milk is natural.
	NFRM3	Raw milk is unprocessed.
	NFRM4	Raw milk can be quickly bought after being milked.
Reusable Raw Milk Bottle (RRMB), adapted from [98,99]	RRMB1	The raw milk packaging in the vending machine is reusable and does not pollute the environment.
	RRMB2	Since I can reuse the packaging, I do not pay extra for it.
	RRMB3	The farmer receives a fair price for the raw milk delivered to the vending machines.
Raw Milk (RM), adapted from [51,100]	RM1	I prefer to drink machine-wheat milk because I can process it myself.
	RM2	I prefer fresh milk from vending machines because it is raw (unprocessed).
	RM3	I prefer raw milk from the vending machines because it is safe.
Raw Milk Knowledge Provenance (RMKP), adapted from [13,101]	RMKP1	I know which farm the raw milk comes from.
	RMKP2	I can visit the farm from which the raw milk comes.
Willingness to Buy Raw Milk (WBRM), adapted from [89]	WBRM1	I am willing to purchase raw milk from vending machines.
	WBRM2	In the future, I will buy raw milk from vending machines more often.
	WBRM3	In the future, I will purchase raw milk from vending machines.
Raw Milk Image (RMI), adapted from [88]	RMI1	Buying raw milk is attractive.
	RMI2	Buying raw milk is a correct decision.
	RMI3	I am happy with the raw milk purchased.
	RMI4	I am satisfied with the raw milk that I purchased.
	RMI5	The raw milk purchased is a safe product.

Source: own research.

### 3.2. Research Sample

Quantitative-based exploratory research was implemented during 2022 among Romanian consumers of different ages and sex (see Table 2). Most of the respondents who buy raw milk from vending machines have higher education (64.5% of the respondents) and were between 18 and 30 years (32.3%).

### 3.3. Statistical Analysis

A model estimation with the help of partial least squares-based structural equation modeling was performed in SmartPLS 3.0 [102] (see Figure 1). To estimate the data, a two-step approach was followed. In the first phase, the measurement model was assessed. This allowed us to determine the reliability and validity of the measures. In the second phase, the relationships among the latent constructs were validated. In the third phase, the authors relied on confirmatory factor analysis. This allowed for the assessment of the validity and reliability of the outer model. The results suggest that the model has internal consistency (Table 3) as the threshold for the loadings, but also the Cronbach’s alpha for the constructs is fulfilled (>0.7) [94,103]; the extracted average variance (AVE) and the composite reliability (CR) also fulfill the threshold (>0.5) [94,104].

Furthermore, we also relied on the Fornell–Larcker [105] criterion (Table 4). Interitem collinearity with the variance inflation factor (VIF) was tested. The VIF values range between 1.341 and 3.125, so the recommended threshold of 5 is met [106]. To assess the multicollinearity of the inner model, the VIFs were also computed. As the highest value of 1.633 is below 3.3 (CNM→MI), there is no multicollinearity between the constructs.

## 4. Results and Discussions

**Figure 2:**
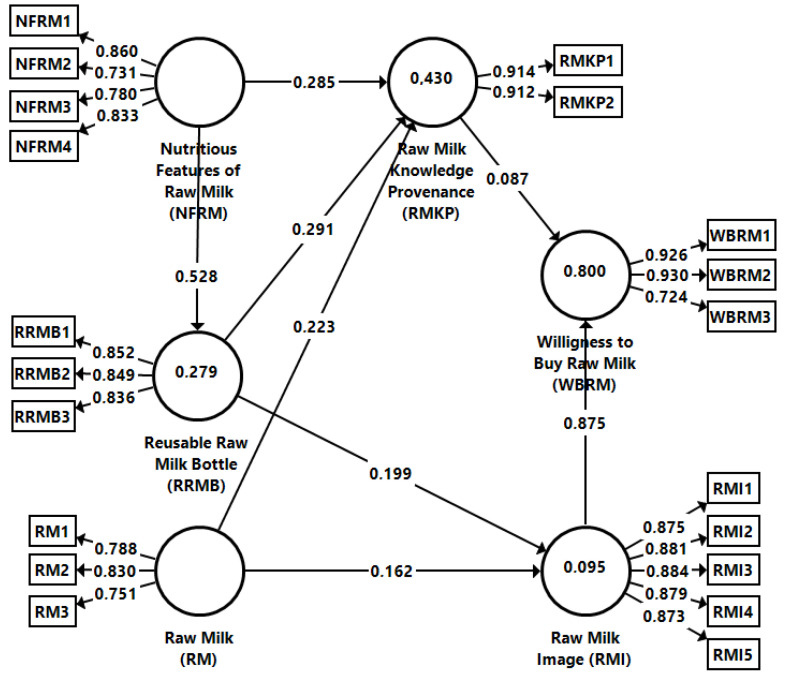
Path coefficients and estimates of the research model. Source: own research.

First, the relationships among the latent variables were assessed using a bootstrap procedure. Using the t-statistics, the hypotheses could be accepted (Table 5). The model is acceptable, as the squared root mean residual (SRMR) has a value of 0.062 (<0.08) for the saturated model and 0.067 (<0.08) for the estimated model. Nutritious features of raw milk explains 27.8% of the variance of reusable raw milk bottle (R^2^ = 0.278); nutritious features of raw milk, reusable raw milk bottle, and raw milk explain 43.0% of the variance in the provenance of milk knowledge (R^2^ = 0.430); and nutritious features of raw milk, reusable raw milk bottle, and raw milk explain 9.5% of the variance in the raw milk image (R^2^ = 0.095). Raw milk image and raw milk knowledge explain 80% of the variance in the willingness to buy milk (R^2^ = 0.800), thus defining a strong predictive power of the structural model (Figure 2).

The first hypothesis (H1) assumes that nutritious features of raw milk correlates with reusable raw milk bottle. The results (β = 0.528; T-value = 12.300; *p* < 0.001) show that this correlation is, indeed, strong and positive; therefore, H1 can be accepted. Similar results are also highlighted in the literature [107], which show that consumer tendency to reuse packaging is influenced by the nutritional characteristics of the products. Resorting to reusable packaging for raw milk packaging increases consumer interest, and consumers’ are willing to pay up to 20% more for milk packaged this way [87]. 

However, other studies also confirm the tendency of consumers to base their milk purchase decisions on its nutritional qualities, as well as affordability, related to sustainability elements [87,108,109]. In contrast to our study, other researchers have considered more the influence of sociodemographic characteristics on packaging reuse and less on the intrinsic characteristics of the products. Gender is a discriminatory element when it comes to packaging reuse, with women paying more attention to aspects of packaging sustainability than men [110].

People’s willingness to pay more for reusable packaging depends, according to Baird et al. [111], on individual, motivational, and contextual variables. Furthermore, according to [87], consumers will only choose sustainable milk packaging when it does not contrast with the very high price. Thus, even if the product has excellent nutritional qualities and its packaging is sustainable, the price can be a barrier to the decision to buy the product.

The second hypothesis presumes that the nutritious characteristics of raw milk exert a positive correlation on the provenance of knowledge about raw milk. The results (β = 0.285; T-value = 5.604; *p* < 0.001) confirm the positive correlation between the concepts, so H2 can also be accepted. The results in [112] show that products with a known provenance are valued by consumers through factors such as convenience and sensory features. Contrary to our findings, [87] shows that economic and environmental sustainability benefits exert a positive influence on the local sourcing of milk. However, [87] confirms the importance of milk provenance (i.e., local origin) in the purchasing process, as it is one of the top three factors in the choice of milk, along with the expiration date and food safety. Consumers often pay attention to the territorial origin of milk [113,114].

The third relationship theorizes that the reused raw milk bottle exerts a positive correlation on the provenance of raw milk knowledge. The results (β = 0.291; T-value = 5.974; *p* < 0.001) prove the strong positive and significant relationship; therefore, the hypothesis H3 can be accepted. Consumers are willing to pay more for packaged milk using biodegradable materials, regardless of its provenance [115]. The importance of knowing the origin of milk is also highlighted in previous research [116], which shows that consumer choices on buying milk are based on attributes, as well as extrinsic attributes. Thus, the origin of the product, its brand, and local origin are among the most important attributes.

The next hypothesis investigates the correlation between reused raw milk bottles and raw milk image. In this case, the results (β = 0.199; T-value = 3.849; *p* < 0.001) confirm that there is, in fact, a strong and positive relationship between these constructs, which also allows us to accept H4. Since this relationship has not been empirically investigated in the previous literature, we consider our result to be particularly important, highlighting the impact of packaging on generating the image of raw milk in the consumer’s mind.

The fifth hypothesis studies the impact of the safe characteristics of raw milk on the provenance of raw milk knowledge. In this case, the results (β = 0.223; T-value = 4.554; *p* < 0.001) confirm a strong and positive correlation, so H5 is confirmed. Our results confirm previous findings [117] that identified food safety as being associated with the origin of the product; consumers consider domestic products to be safer than industrial ones [75]. H6 assumes that there is a positive correlation between raw milk features and raw milk image. In this case, the results (β = 0.162; T-value = 3.069; *p* < 0.005) confirm this correlation, so this hypothesis can also be accepted. The results obtained are contrary to a recent study [118] that argues that food safety is less important for consumers who purchase local products, as they have greater trust in local products.

The seventh hypothesis presumes that the provenance of raw milk knowledge has a positive correlation on consumer willpower to buy raw milk. This positive correlation was also confirmed by the results (β = 0.087; T-value = 4.070; *p* < 0.001), although the intensity of the relationship is lower. Our results are supported by the literature [119], which shows that some consumers’ purchase decisions are influenced by knowledge of the provenance of their preferred foods so H7 can be accepted. The last hypothesis investigates the impact of raw milk image on consumers’ willingness to buy raw milk. The results (β = 0.875; T-value = 73.651; *p* < 0.001) confirm the strong positive correlation, so H8 is accepted. The literature [112] has shown that foods with a known provenance are associated with a positive image of the product, as well as with an additional motivation to purchase local products, which confirms our findings. 

As consumers are currently more interested in the product itself than the raw milk provenance or the conditions of obtaining it at the farm, it is necessary to raise awareness of the fact that, in order to obtain a quality product, the farm must comply with animal welfare conditions, fodder quality, location in a certain environment, hygiene of facilities, and a competent human factor, all of which directly influence the intrinsic qualities of the product and are, therefore, image vectors for the safety of the purchased products. The development of a personal connection with the farmer or farm employees contributes to the social and financial strengthening of local communities, and it also offers the consumer the opportunity to purchase other products that the producer can make available.

A limitation of this research regards the fact it surveyed consumers who are familiar with this type of vending machine, that is, those who regularly buy raw milk from vending machines. Future research can draw comparisons between consumers who use these raw milk vending machines and those who buy milk from retail chains. It can also propose comparative analyses among consumer perceptions of milk sold at vending machines, milk distributed directly from the farm’s and/or farmer’s agricultural markets and milk sold through retail chains. Another limitation of our research is related to the convenience sampling. Future research can rely on a more representative sample; however, it is difficult to determine the exact number of consumers who buy, on a regular basis, milk from vending machines. One more limitation is the fact that we used a survey. Future studies can employ an experiment with a cross-sectional design, which would allow to measure, at the same time, the exposures and outcomes of the participants [120]. Thus, it would also be interesting to study the differences in the perceptions of urban versus rural consumers towards milk sold through milk dispensers or between large and small urban areas. 

Another interesting analysis could relate to the preference for organic versus conventional food. As the consumption of raw milk from vending machines has the potential to increase factors that cause some consumers to avoid the consumption of products from vending machines, despite their advantages, this can also be evaluated. Some of these factors could be the appearance of the machine or the convenience of multiple purchases offered by supermarkets. The image of the product closely correlates with the desire to purchase this type of product, so the research should be extended to other factors that could influence this image.

## 5. Conclusions

From a theoretical perspective, this paper extends studies focused on the stimulus–organism–response model, highlighting how it can be transposed for food research. Image is a particularly important factor in the raw milk purchasing process. Consequently, to stimulate the desire to purchase it, communication with the customer must be increased by promoting the factors that contribute to the formation of this image: product safety, packaging recycling, and ecological aspects linked to production and distribution but also aspects linked to the nutritional qualities of unprocessed milk.

From a managerial perspective, this article highlights the possibilities of expanding raw milk distribution, with vending machines being a viable strategy, at least for local farmer associations, through which minimally processed milk can reach consumers safely and hygienically. Furthermore, it is clear from the research that there is a need to develop a favorable mentality among consumers towards purchasing natural and minimally processed milk. In fact, many consumers prefer to buy milk from stores because it comes ready-packed in containers that can be easily disposed of and washing is not necessary. However, consumer awareness of the negative impact of packaging on pollution and recycling costs should make it easier for consumers to buy food that can be packaged in reusable containers. In response to the pressures generated by large food supply chains, farmers should make consistent efforts to develop short food chains, such as farm gate sales, sales in street markets or farmers’ markets, use of collective supply systems, ‘box shopping systems’, and vending machines to serve customers.

## Figures and Tables

**Table 2:** Sociodemographic characteristics of the respondents.

Demographics (N = 322)		Frequency	Relative Frequency %
Age	18–30	104	32.3
	31–40	77	23.9
	41–50	88	27.3
	51–60	23	7.1
	Over 60	30	9.3
Gender	Male	90	27.9
	Female	232	72.1
Size of town	Under 50,000 inhabitants	71	22.0
	Over 50,000 inhabitants	251	78.0
Average net monthly income	Under RON 1500 (EUR 300)	114	35.4
	RON 1501–2500 (EUR 301–500)	134	41.6
	More than RON 2500 (over EUR 500)	61	18.9
Number of children (under 18 years)	One	106	32.9
	Two	63	19.6
	Three or more	153	47.6
Education level	Professional school	40	12.4
	High school	75	23.3
	Higher studies	207	64.5

Source: own research.

**Table 3:** Loadings of items, validity, and reliability analysis.

Item	Loading	Cronbach’s Alpha	AVE	CR
NFRM1	0.860	0.815	0.644	0.878
NFRM2	0.731			
NFRM3	0.780			
NFRM4	0.833			
RRMB1	0.852	0.802	0.715	0.883
RRMB2	0.849			
RRMB3	0.836			
RM1	0.788	0.704	0.625	0.833
RM2	0.830			
RM3	0.751			
RMKP1	0.914	0.800	0.833	0.909
RMKP2	0.912			
WBRM1	0.926	0.830	0.749	0.898
WBRM2	0.930			
WBRM3	0.724			
RMI1	0.875	0.926	0.771	0.944
RMI2	0.881			
RMI3	0.884			
RMI4	0.879			
RMI5	0.873			

Item loading > 0.7; Cronbach’s alpha > 0.7; AVE > 0.5; CR > 0.7 [94,103,104].

**Table 4:** Discriminant validity analyses.

Construct	NFRM	RMI	RMKP	RRMB	RM	WBRM
NFRM	0.802					
RMI	0.212	0.878				
RMKP	0.558	0.181	0.913			
RRMB	0.527	0.272	0.542	0.846		
RM	0.533	0.251	0.506	0.451	0.790	
WBRM	0.261	0.890	0.245	0.273	0.248	0.865

CNRM: Nutritious features of raw milk; RMI: raw milk image; RMKP: raw milk knowledge provenance; RRMP: reusable raw milk bottle; RM: Raw Milk; WBRM: willingness to buy raw milk. The value of AVE for each latent variable is higher than the correlation coefficient between the competent variables and all the different variables. Source: own research.

**Table 5:** Path coefficients of the structural equation model.

Path	Path Coefficient	Standard Deviation	T-Value	*p*-Value	CI ^1^	Hypothesis
NFRM→RRMB	0.528	0.043	12.300	0.000 ***	0.442–0.618	H1-Supported
NFRM→RMKP	0.285	0.051	5.604	0.000 ***	0.185–0.389	H2-Supported
RRMB→RMKP	0.291	0.049	5.974	0.000 ***	0.191–0.384	H3-Supported
RRMB→RMI	0.199	0.052	3.849	0.000 ***	0.093–0.301	H4-Supported
RM→RMKP	0.223	0.049	4.554	0.000 ***	0.130–0.318	H5-Supported
RM→RMI	0.162	0.053	3.069	0.003 **	0.050–0.280	H6-Supported
RMKP→WBRM	0.087	0.021	4.070	0.000 ***	0.049–0.123	H7-Supported
RMI→WBRM	0.875	0.012	73.651	0.000 ***	0.851–0.900	H8-Supported

** *p* < 0.01; *** *p* < 0.001. NFRM: nutritious features of raw milk; RMI: raw milk image; RMKP: raw milk knowledge provenance; RRMP: reusable raw milk bottle; RM: raw milk; WBRM: willingness to buy raw milk. ^1^ CI = confidence interval (2.5% to 97.5%). Source: own research.

## Data Availability

The data presented in the study are available on request from the corresponding author.
